# Heterogeneity in COVID-19 pandemic-induced lifestyle stressors predicts future mental health in adults and children in the US and UK

**DOI:** 10.1016/j.jpsychires.2021.12.058

**Published:** 2022-01-03

**Authors:** Aki Nikolaidis, Jacob DeRosa, Mirelle Kass, Irene Droney, Lindsay Alexander, Adriana Di Martino, Evelyn Bromet, Kathleen Merikangas, Michael Peter Milham, Diana Paksarian

**Affiliations:** a Child Mind Institute, United States; b Stony Brook University, United States; c National Institute of Mental Health, United States

## Abstract

**Introduction:**

Identifying predictors of mental health symptoms after the initial phase of the pandemic may inform the development of targeted interventions to reduce its negative long-term mental health consequences. In the current study, we aimed to simultaneously evaluate the prospective influence of life change stress, personal COVID-19 impact, prior mental health, worry about COVID-19, state-level indicators of pandemic threat, and socio-demographic factors on mood and anxiety symptoms in November 2020 among adults and children in the US and UK.

**Methods:**

We used a longitudinal cohort study using the Coronavirus Health Impact Survey (CRISIS) collected at 3 time points: an initial assessment in April 2020 (“April”), a reassessment 3 weeks later (“May”), and a 7-month follow-up in November 2020 (“November”). Online surveys were collected in the United States and United Kingdom by Prolific Academic, a survey recruitment service, with a final sample of 859 Adults and 780 children (collected via parent report). We found subtypes of pandemic-related life change stress in social and economic domains derived through Louvain Community Detection. We assessed recalled mood and perceived mental health prior to the pandemic, worries about COVID-19, personal and family impacts of COVID-19, and socio-demographic characteristics. We used a conditional random forest approach to predict November mood states using these data from April and May and to rank the variable importance of each of the predictor items.

**Results:**

Levels of mood symptoms in November 2020 measured with the circumplex model of affect. We found 3 life change stress subtypes among adults and children: Lower Social/Lower Economic (adults and children), Higher Social/Higher Economic (adults and children), Lower Social/Higher Economic (adults), and Intermediate Social/Lower Economic (children). Overall, mood symptoms decreased between April and November 2020, but shifting from lower to higher-stress subtypes between time points was associated with increasing symptoms. For both adults and children, the most informative predictors of mood symptoms in November identified by conditional random forest models were prior mood and perceived mental health, worries about COVID, and sources of life change.

**Discussion:**

The relative importance of these predictors was the most prominent difference in findings between adults and children, with lifestyle changes stress regarding friendships being more predictive of mood outcomes than worries about COVID in children. In the US, objective state-level indicators of COVID-19 threat were less predictive of November mood than these other predictors. We found that in addition to the well-established influences of prior mood and worry, heterogeneous subtypes of pandemic-related stress were differentially associated with mood after the initial phase of the pandemic. Greater research on diverse patterns of pandemic experience may elucidate modifiable targets for treatment and prevention.

## 1. Introduction

As the COVID-19 pandemic has led to worldwide illness, death, and disruptions in daily life, its effects on emotional well-being have become a public health priority (Kantor and Kantor, 2020). Overall, studies have demonstrated high levels of anxiety and related conditions in samples of adults (Fitzpatrick et al., 2020; Kantor and Kantor, 2020), with fewer investigations in children and adolescents (Duan et al., 2020). Most studies have been cross-sectional, and many have focused on at-risk subgroups (e.g., healthcare and essential workers; (Demirjian et al., 2020). Longitudinal studies of general population samples have found that mental health problems initially increased compared to pre-pandemic levels (Li et al., 2020; Pierce et al., 2020; Westrupp et al., 2020), followed by a decrease as the pandemic continued (Hawes et al., 2021; Pierce et al., 2021; Robinson and Daly, 2020). This is consistent with prior research on population mental health responses to disaster, which has shown that while transient increases in symptomatology are common, long-lasting problems are rare (Goldmann and Galea, 2014). Longitudinal studies that identify predictors of poor mental health versus resilience after the initial phase of the pandemic could help to inform the development of targeted interventions to reduce long-term mental health consequences.

Prior disaster mental health research has consistently found that pre- disaster mental health and the degree of fear and worry during a disaster are primary determinants of mental health outcomes (Whalley and Brewin, 2007). Mental health responses to disasters are also worse among those who are directly and severely impacted, and vary by socio-demographic characteristics such as younger age, gender, lower income, and minority race/ethnicity (Bonanno et al., 2007). Consistent with these findings, initial work by our team and others highlighted prior mental health and the degree of worry about COVID-19 as key predictors of mood in the initial months of the COVID-19 pandemic (Ahorsu et al., 2020; Fitzpatrick et al., 2020; Nikolaidis et al 2020). Along with others, our prior work also highlighted the importance of the widespread changes in daily life and accompanying stressors induced by the pandemic for mental health (Breslau et al., 2021; Fancourt et al., 2021; Kelly, 2020; Nikolaidis et al., 2020; Pan et al., 2021; Robinson and Daly, 2020; Serafini et al., 2020). Together these prior findings underscore the multifactorial influences of the pandemic among families and individuals. A greater understanding of the long-term role of pandemic-related life change stress, in relation to other risk factors for poor mental health, is needed in order to understand potential avenues for intervention.

The goal of the current study was to extend our previous findings by using a longitudinal design to characterize the role of life change stress in shaping mood after the initial phase of the COVID-19 pandemic. After an initial assessment in April 2020 (“April”), we reassessed participants three weeks later (“May”) to capture rapid changes during the beginning of the pandemic, and then conducted a 7-month follow-up in November 2020 (“November”) among our cross-national sample of adults and children (assessed via self and parent report, respectively) in the US and UK. Our aims were to: 1) Define subtypes of life change stress at 3 weeks and their associations with mood and other correlates. 2) Assess the stability of subtype membership across time points, and how subtype transitions were associated with changes in mood and worries about COVID-19. 3) Identify the importance of life change stress subtypes in predicting mood at follow up, while considering other factors including demographics, recalled prior mood and mental health, worries about COVID-19, personal and family pandemic impact, and objective state- level indicators of pandemic threat. To our knowledge, this is the first longitudinal study to simultaneously investigate the roles of life change stresses, personal COVID-19 impact, prior mental health, worry about COVID-19, state-level indicators, and socio-demographic factors in levels of mood among adults and children in multiple countries.

## 2. Methods

### 2.1. Samples

Data were collected through Prolific Academic (PA; https://www.prolific.ac/ ), an online crowdsourced survey recruitment service (Table 1). Participants who signed up to join the PA participant pool received monetary compensation for their time. PA offers samples that broadly reflect the population distributions of age, sex, and race in the US and UK; participants have been shown to be more diverse and provide higher quality data than similar data collection platforms (Peer et al., 2017). In March 2020 we requested four samples of 1500 participants from the US and UK, both adult self-report and parent report. Portions of the sample were targeted at regions that were more severely impacted by COVID-19 in late March 2020 (New York, California, London, and Manchester). For the child (parent report) sample, users were screened based on having a child between 5 and 18 years old and were asked to report on their oldest child in that age range; reports received for children over age 25 were excluded. No additional exclusion criteria were given to PA.

Three waves of data were collected in 2020: at baseline (April 7th–17th), 3 weeks (April 24th-May 4th), and 7-month follow up (October 31st-November 23rd). For the present analyses we excluded participants with uncompleted forms and incomplete information for subtyping (described below). Final analytic sample sizes were: 3259 in April (1793 adults and 1466 parents), 2553 at 3 weeks (1380 adults and 1173 parents), and 1639 in November (859 adults and 780 parents).

Exemption from IRB oversight was approved by the Advarra Institutional Review Board. PA participants were required to agree to PA’s Terms of Service notification (https://prolific.ac/assets/docs/Participant_Terms.pdf ) to complete surveys. Per the IRB exemption, no additional informed consent was required. Comparisons between the demographics of the included sample and both the drop out and Census for US and UK are included in the Supplement (eTable2, eTable3).

### 2.2 Measures

The Coronavirus Health Impact Survey (www.crisissurvey.org) was administered online via REDCap software at all 3 time points. Abbreviated baseline versions of CRISIS v0.3 were administered in April and follow-up versions of CRISIS v0.3 were administered in May. The CRISIS v0.3 baseline forms were revised and expanded for the November follow-up (available by request).

#### 2.2.1. Mood

Ten items from the circumplex model of affect (Larsen and Diener, 1992; Posner et al., 2005; see Supplement) rated on a 5-point scale were included to measure mood. Prior mood was assessed at baseline by asking participants to recall the 3 months prior to the pandemic. Mood over the past 2 weeks (“Current mood”) was included from the May and November time points. Based on excellent unidimensional fit (see eTable4), items were averaged to generate a total score (range: 1 to 5), with higher scores indicating worse mood. Unadjusted associations with mood are included in the Supplement (eTable5, eTable6).

#### 2.2.2. Predictors

*Participant characteristics:* Age, sex, self- or parent-rated mental and physical health, urbanicity, informant education, and employment (adults only) were reported on the CRISIS in April. Race was reported to PA and combined with CRISIS information on Hispanic ethnicity to generate the following categories: Hispanic, non-Hispanic White, non- Hispanic Black, Asian, and Other race/ethnicity. For children, parent race was used as a proxy for child race. Perceived mental health prior to the pandemic, and physical health during the pandemic, were rated on 5-point scales from “excellent” to “poor.”

##### Personal COVID-19 impact:

Perceived exposure to SARS-CoV-2, COVID-19 symptoms, family member diagnosis of COVID-19, and impacts on family members such as hospitalization, quarantine, and job loss because of COVID-19 were reported on the CRISIS in May with respect to the past 2 weeks. In April, participants reported whether they or a household member were an essential worker.

##### COVID-19 Worries in the past two weeks:

In May, participants reported on a five-point Likert scale how worried they have been during the past two weeks about infection, friends and family being infected, and possible impacts on physical and mental health, as well as time spent reading or talking about COVID-19 and hope that the pandemic will end soon. Based on psychometric analyses (described in Supplement), these were averaged to generate a total score (range: 1 to 5; hereafter referred to as “COVID-19 Worries”).

##### Life Changes due to the pandemic in the past two weeks:

Downstream and subjective impacts of structural changes, such as changes in social contacts, effects on family relationships, changes in living situation, food insecurity, and stressors associated with these changes were reported on the CRISIS (14 items; see Supplement). Life change stress subtypes were derived independently at all 3 timepoints based on these items (described below).

##### Objective Government Response Tracker (OxCGRT) Indices:

Following the model of Vibert et al., (in prep), state-level indices from OxCGRT (Hale et al., 2021) were used to indicate objective levels of COVID-19 threat in each US participant’s state. Specifically, we included: Containment and Health Index (lockdown restrictions and closures related to COVID-19), Economic Support Index (economic relief for households provided by the government), Confirmed Cases by state per 1,000,000 residents, and Confirmed Deaths by state per 1,000,000 residents (see Supplemental Methods). These indices covered the time period: January 1st, 2020 to March 19th, 2021. Average values of each index were calculated from April 1st to May 31st. Regionally specific containment data were not available for the UK, and so our analyses using containment included only US data.

### 2.3. Analysis

#### Community Detection Based Subtyping (Aims 1 & 2):

**Fig. 1.**
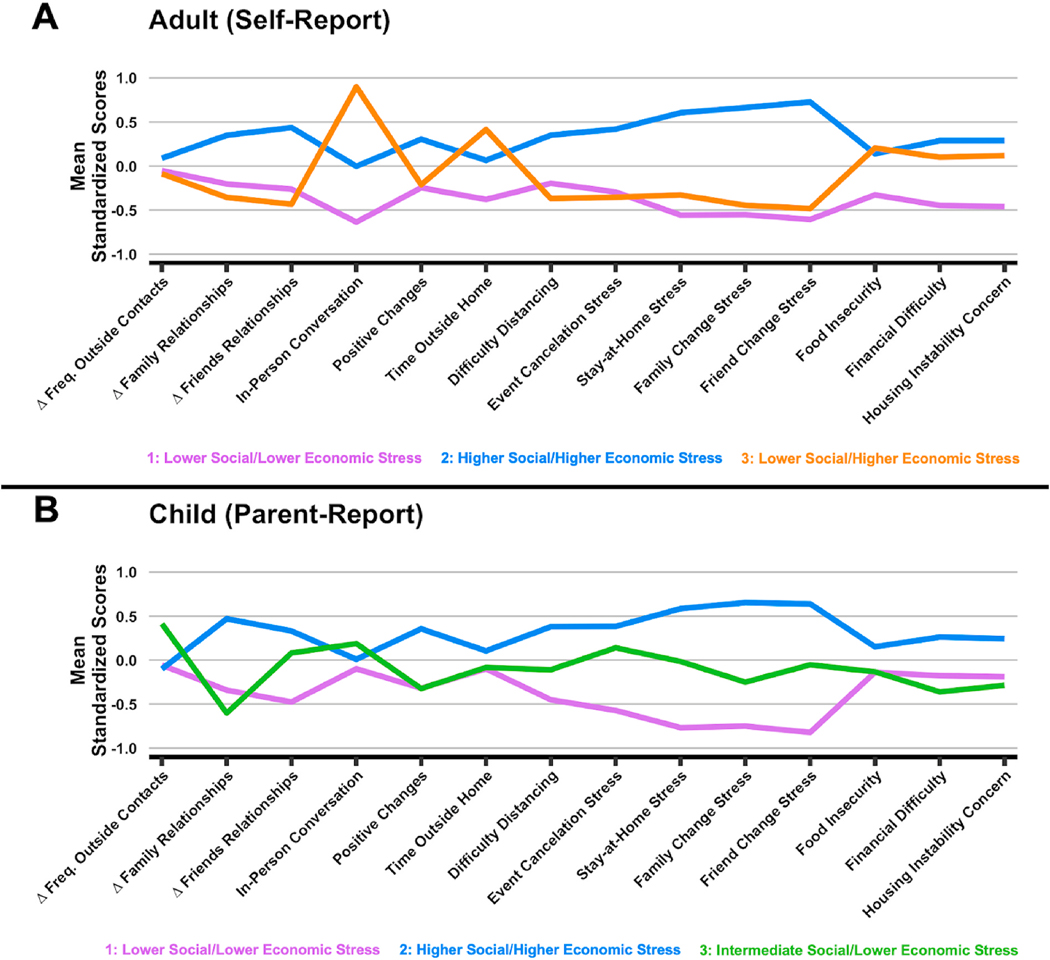
May Life Change Stress Subtypes. A. Life Change Stress Subtype profiles for adults (top) and children (bottom) in May 2020. Mean normalized profile loadings are displayed on the y-axis. Δ Family Relationships and Δ Friends Relationships are coded so that higher scores indicate worsening quality of relationships. In-Person Conversation, Positive Changes, and Time Outside Home are coded so that higher scores indicate less conversations, positive changes, and time spent outside. Adult Report: Purple (1): Lower Social/Lower Economic Stress, Blue (2): Higher Social/Higher Economic Stress, Orange (3): Lower Social/Higher Economic Stress. Parent Report: Purple (1): Lower Social/Lower Economic Stress, Blue (2): Higher Social/Higher Economic Stress, Green (3): Intermediate Social/Lower Economic Stress.

**Fig. 2.**
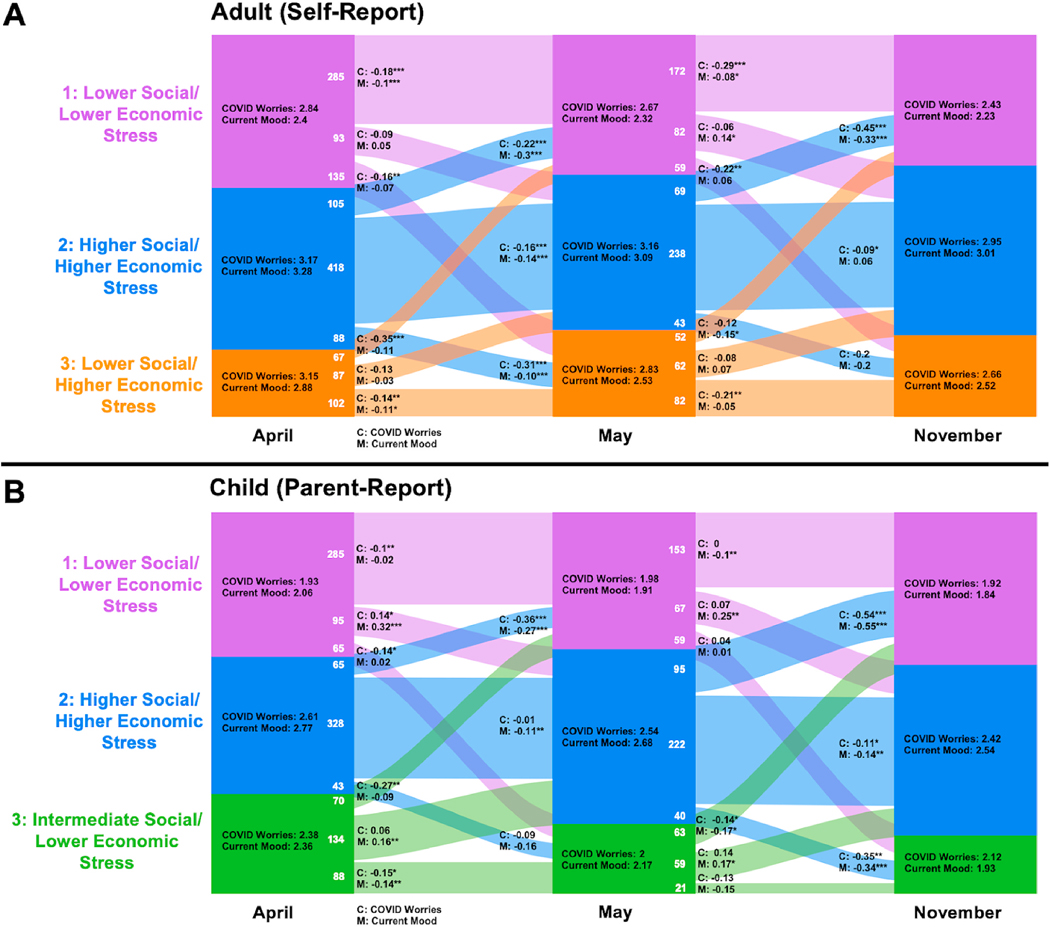
Stability and change in Life Change Stress Subtype across time points with mean mood and COVID Worries scores by group. Note: Colored areas correspond to the proportion of participants in each subgroup at each time point. The numbers of individuals moving between Subtypes are given in white, with proportions indicated by the width of the colored paths. Mean COVID Worries and Current Mood scores are represented by Subtype in the center of each column. COVID Worries (C) and Mood (M) are represented in the change paths for each change group. T-tests compared the change in the scores between time points for each change group: * = p < .05, ** = p < .01, *** = p < .001.

Life change stress subtypes were derived using Louvain community detection (Aim 1; LCD; Fig. 1; eFigure1; see Supplemental Methods), enhanced through boot-strap aggregation (i.e., bagging), as in our prior study (Nikolaidis et al., 2020). Structural similarity across samples and timepoints was confirmed using Pearson’s correlation. LCD is a clustering technique that selects clusters to maximize within-cluster coherence and between-cluster segregation. Subtype differences between individual items are described in the supplement (eTable7). We compared demographic characteristics and COVID-19 impact indicators across May subtypes using one-way ANOVA (Table 2; eTable8). We also estimated mean mood and COVID-19 Worries scores according to patterns of change in subtype membership across the 3 timepoints and used t-tests to test mean differences (Aim 2; Fig. 2).

#### Conditional Random Forest (Aim 3):

Finally, we used conditional random forest (CRF) to predict mood in November based on prior mood and perceived mental and physical health, COVID-19 worries factor scores, May life change stress subtype, OxCGRT indices, socio-demographic characteristics, and personal and family COVID-19 impact. Random forest (RF) is a robust predictive machine learning technique known for its ability to handle dependencies and interactions between predictor variables (See Supplemental Methods; (Breiman, 2001; Probst et al., 2019)). CRF overcomes some limitations of traditional RF and estimates how much each variable contributes to overall model accuracy, conditioned on other included variables (Strobl et al., 2007). We built 3 sets of CRF models: a base model including all predictors described above, using life change stress subtypes; a second model which replaced life change stress subtypes with their constituent items; and a third model in the US sample alone which added the OxCGRT indices.

Because our prior study demonstrated highly reproducible findings across the US and UK, and to maximize power for prediction modeling, analyses were conducted combining the US and UK samples. The proportion of missing data in the analytic sample was less than 2% for all variables and missing data were handled via model-wise deletion.

## 3. Results

### 3.1. Sample characteristics

Table 1 shows demographic characteristics and COVID-19 impact indicators for the November 2020 sample, along with means and standard deviations of November mood scores. The majority of adults were females (57.6%), white (75.1%), and aged 30–39 (24.7%), while the majority of children were males (53.6%), white (81.5%), and aged 6–13 years (60.3%). Overall, mood symptom and COVID-19 Worries levels decreased between the April and November both in adults (mood: 0.13; p < .0001; COVID-19 Worries: 0.35; p < .0001) and children (mood: 0.17; p < .0001; COVID-19 Worries: 0.16; p < .0001). Differences in November mood by participant characteristics are displayed in Table 1. Mean COVID-19 Worries and prior mood scores by demographics and COVID-19 impact indicators are presented in eTables5 and 6.

### 3.2. Life change stress subtypes (Aim 1)

Life change subtypes derived from the 3-week time point (Fig. 1) were named based on relative ratings of social/interpersonal and economic stressors (See eTable7 and supplemental results). Adult subtypes were: *Lower Social/Lower Economic stress* (purple; 1), *Higher Social/ Higher Economic stress* (blue; 2), and *Lower Social/Higher Economic stre*ss (orange; 3). Among children, subtypes were: *Lower Social/Lower Economic stress* (purple; 1), *Higher Social/Higher Economic stress* (blue; 2), and *Intermediate Social/Lower Economic stress* subtype (orange; 3).

### 3.3. Longitudinal subtype change (Aim 2)

Fig. 2 shows the stability of, and change in, subtype membership across time points, with accompanying mean scores for COVID-19 Worries and mood. Most participants stayed in the same subtype across time points. Specifically, 805/1380 adults and 747/1173 children remained stable between April and May, and 492/859 adults and 396/ 780 children remained stable between May and November. Subtype 3 (Lower Social/Higher Economic for adults and Intermediate Social/ Lower Economic for children) appeared the least stable. We saw an overall trend of either decrease or no change in COVID-19 Worries and mood across timepoints and subtypes for both adults and children. The only significant increase among adults was in mood for those who moved from the Lower Social/Lower Economic subtype in May to the Higher Social/Higher Economic subtype in November (estimate = 0.14, p < .05). More increases were seen among children. Children who changed from Lower Social/Lower Economic to Higher Social/Higher Economic showed increases in COVID Worries (April–May only; estimate = 0.14, p < .05) and mood (April–May: estimate = 0.32, p < .001; May–November: estimate = 0.25, p < .01). Children who moved from Intermediate Social/Lower Economic to Higher Social/Higher Economic showed increases in mood symptoms across both transitions (April–May: estimate = 0.16, p < .01; May–November: estimate = 0.17, p < .05).

### 3.4. Predicting November mood (Aim 3)

**Fig. 3.**
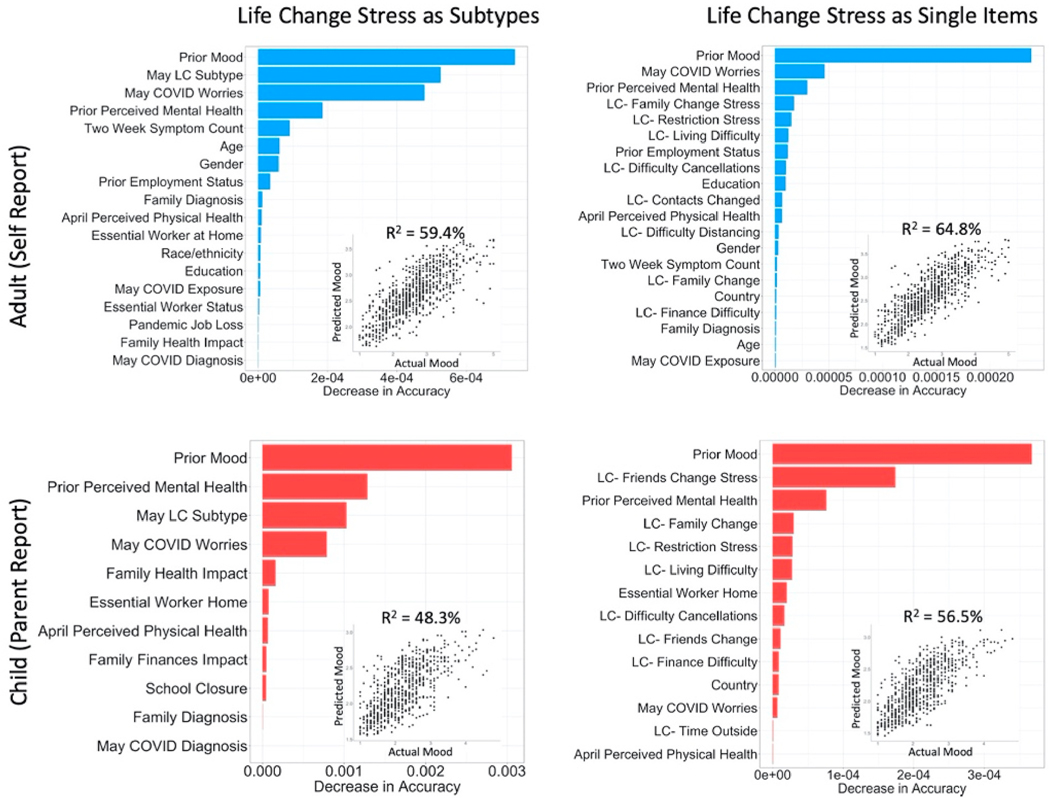
Results of conditional random forest models predicting Current Mood in November 2020. Note: Conditional Random Forest Models in adults (top; n = 827) and children (bottom; n = 750) showing the conditional variable importance of each predictor. Variable importance is calculated based on decreases in accuracy when a variable is removed conditioned on all other variables in a model using balanced bootstrapped partitions of the data to ensure unbiased importance estimates. Only variables with importance values greater than zero are shown. Life change stress was included as data-derived subtypes on the left and as individual indicators on the right.

Conditional random forests including participant characteristics, COVID-19 impact indicators, prior mood and mental health, COVID-19 worries, and life change subtypes predicted substantial variation in November mood (Fig. 3; Adult R^2^ =59.4%; Child R^2^ =47.8%). The most important predictors were prior mood, COVID-19 Worries, prior perceived mental health, and life change subtype for adults and children. While COVID-19 Worries was the second most informative predictor for adults, life change subtypes were the second most informative for children. When life change stress was included as individual items, social/interpersonal stress items (such as family change stress, friend change stress, and family change) showed the highest conditional variable importance for both adults and children, and the variance explained increased (Fig. 3; Adult R^2^_+_5.4%; Child R^2^_+_8.6%). For the US samples of adults and children, adding indicators of US state-level COVID-19 threat from OxCGRT (eFigure2) modestly increased variance explained, but these variables exhibited lower conditional importance (Adult R^2^_+_3.1%; Child R^2^_+_1.1%) relative to other variables.

## 4. Discussion

In a longitudinal study covering the beginning of the COVID-19 pandemic and a six month follow-up between April and November 2020, we found that prior mood, worry about COVID-19, and profiles of pandemic-induced life change stress were the most important predictors in April and May of future mood symptom levels in November 2020. The importance of prior mood and worry about COVID-19 is supported by extensive prior studies of mental health during the pandemic (Breslau et al., 2021; Holman et al., 2020; O’Connor et al., 2021; Pan et al., 2021), as well as studies following other large scale traumatic events (i. e., natural disasters, terrorist attacks, nuclear reactor meltdowns; Bromet and Havenaar, 2007; Freedy et al., 1993; Keller et al., 2012; Whalley and Brewin, 2007). Of note, the relative importance of these three predictors was the most prominent difference in findings between adults and children, with lifestyle changes stress (particularly that related to friendships) being more predictive of mood outcomes than worries about COVID-19 in children. Country did not emerge as an important predictor of later mood, implying similarity of predictors across sites. The importance of the life change stress subtypes underscores the heterogeneity in the effects of the pandemic on people’s lives.

In the current study, patterns of life change stress were highly reproducible across samples and over time. They largely captured individual variation in secondary effects of the pandemic beyond medical illness, such as changes in social relationships and financial security. Their importance is consistent with other studies of the COVID-19 pandemic, which have found associations of social distancing, stay-at- home orders, and financial strain with mental health (Elbogen et al., 2021; Marroquín et al., 2020; Williams et al., 2021). Our analysis of stability and switching life change stress subtypes indicated that for most groups, mood symptoms decreased between time points. This is consistent with other COVID-19 research showing that mental health symptoms rose at the start of the pandemic and subsequently declined (Fancourt et al., 2021; Robinson and Daly, 2020). Higher mood levels in November may be an indicator of risk for prolonged mental health problems following the pandemic, although this needs to be confirmed with additional follow-up. Groups who deviated from the decreasing pattern were those who changed into a high-stress subtype between study waves, further demonstrating the importance of variation in life change stress as a contributor to mental health. The greater importance of life change stress subtypes over objective state-level indicators of pandemic threat may be because they are more proximally tied to mental health.

Although we cannot infer causal relationships, our longitudinal results may help to inform the targeting of intervention strategies and anticipate health services needs during future crises (Campion et al., 2020). Individuals with higher levels of symptoms prior to the pandemic are at risk of continued poor mental health during the pandemic, and should be regarded as a vulnerable subgroup (Breslau et al., 2021; Druss, 2020; Pan et al., 2021). Research from prior disasters and the current pandemic has demonstrated the importance of reliable information in shaping people’s degree of fear and worry about an event (Bromet and Litcher-Kelly, 2002; Brooks et al., 2020). Therefore, national-level efforts to provide more high-quality information about COVID-19 and increase trust in legitimate information sources may help to decrease mood-anxiety symptoms among the general public. Economic stressors captured in the life change stress profiles may be addressed through policy interventions that decrease housing instability, food insecurity, and economic hardship (Benfer et al., 2021; Leddy et al., 2020). Sources of social and interpersonal stress may vary widely and therefore be more difficult to intervene on from a population level. However, strategies that may be useful include the provision of psychoeducational and coping resources (Bäuerle et al., 2020), implementation of programs to reduce domestic and intimate partner violence and provision of resources for those affected (Evans et al., 2020), and increasing availability of formal and informal mental health services through telemedicine and other alternative venues (Salum et al., 2020; Zhou et al., 2020).

The major limitation of this study is the use of a web-based convenience sample, which raises concerns about the potential for selection biases and may limit the generalizability of our findings. For example, if selection into the sample differed by both mental health and life change stress indicators, this could have inflated the association between life change stress and mood. Use of this sample was motivated by the need to quickly develop and deploy the CRISIS questionnaire. CRISIS being used in several studies across the world will allow for the comparison of results across sample types and locations. In addition, we relied on recall for measurement of pre-pandemic mental health, and because of concern that we would not be able to secure child reports in significant numbers we relied on parent reports for children, which may be less accurate for older children and for internalizing symptoms across ages (Aebi et al., 2017). Furthermore, our estimates may have been influenced by sample attrition over time. Strengths of the study include the cross-national sample that enables us to demonstrate overall consistency of findings across the two geographical locations; the investigation of predictors of mental health of children, which is less common in the COVID-19 literature (Ravens-Sieberer et al., 2021; Wade et al., 2020), and the use of sophisticated analytic techniques that allow us to gauge the relative importance of multiple predictors on mood symptoms.

As vaccination rates increase and society returns to “normal,” the mental health needs of those with continued psychological distress will need to be addressed (Maulik et al., 2020). Longitudinal studies are vital for understanding who is going to continue to struggle post-pandemic. This study suggests that in addition to well-established risk factors for post-disaster mental health, attending to the heterogeneity in the impact of the pandemic on people’s daily patterns of interaction and living may provide useful targets for interventions to reduce mood and anxiety symptom levels.

## Supplementary Material

1

## Figures and Tables

**Table 1:** Sample characteristics with mean Current Mood scores and adjusted associations with Current Mood.

	Adult (Self Report) *N* = 858			Child (Parent Report) *N* = 780		
		
	*n* (%)	Current Mood Mean (SD)	Current M Adjusted	*n* (%)	Current Mood Mean (SD)	Current Mood Adjusted

Country						
US	356 (41.5%)	2.65 (0.8)	0.05	288 (37.0%)	2.21 (0.7)	0.05
UK	502 (58.5%)	2.63 (0.8)	Reference	491 (63.0%)	2.14 (0.7)	Reference
Age						
Under 20	29 (3.4%)	3.00 (0.9)	0.14			
20–29	201 (23.4%)	2.81 (0.8)	− 0.05			
30–39	212 (24.7%)	2.74 (0.8)	Reference			
40–49	138 (16.1%)	2.68 (0.8)	− 0.07			
50–59	130 (15.2%)	2.50 (0.7)	− 0.18^a^			
60 and Over	148 (17.2%)	2.28 (0.8)	− 0.24^b^			
Child Age						
5 and Under				132 (16.9%)	2.13 (0.7)	0.07
6–13				470 (60.3%)	2.19 (0.7)	Reference
14–17				164 (21.0%)	2.16 (0.7)	− 0.11
18 and Over				14 (1.8%)	1.97 (0.7)	−
Gender						
Female	494 (57.6%)	2.76 (0.8)	Reference	360 (46.2%)	2.18 (0.7)	0.03
Male	360 (42.0%)	2.47 (0.8)	− 0.14^b^	418 (53.6%)	2.16 (0.7)	Reference
Other	4 (0.5%)	3.08 (0.3)	−	2 (0.3%)	2.30 (0.1)	−
Race/ethnicity						
Asian	70 (8.2%)	2.64 (0.8)	− 0.15	30 (3.8%)	2.02 (0.5)	− 0.17
Black	51 (5.9%)	2.69 (0.7)	− 0.17	40 (5.1%)	1.79 (0.6)	− 0.36^c^
Hispanic	58 (6.8%)	2.68 (1.0)	− 0.06	46 (5.9%)	2.50 (0.8)	0.32^b^
Other	35 (4.1%)	2.95 (0.8)	0.15	28 (3.6%)	2.12 (0.7)	− 0.15
White	644 (75.1%)	2.62 (0.8)	Reference	636 (81.5%)	2.18 (0.7)	Reference
Urbanicity						
Large City	203 (23.7%)	2.74 (0.8)	0.00	113 (14.6%)	2.21 (0.8)	0.11
Suburbs of a Large City	218 (25.4%)	2.69 (0.8)	0.03	226 (29.2%)	2.18 (0.7)	0.07
Small City	128 (14.9%)	2.62 (0.8)	− 0.09	114 (14.7%)	2.19 (0.6)	0.00
Town or Village	248 (28.9%)	2.58 (0.8)	Reference	273 (35.2%)	2.12 (0.6)	Reference
Rural Area	60 (7.0%)	2.46 (0.8)	− 0.12	49 (6.3%)	2.28 (0.7)	0.16
Informant Education						
Some Grade School	2 (0.2%)	2.45 (0.4)	− 1.16^a^			
Some High School	30 (3.5%)	2.65 (0.8)	− 0.15			
High School Diploma or GED	110 (12.9%)	2.70 (0.8)	− 0.10			
Some College or 2-Year Degree	233 (27.3%)	2.65 (0.8)	− 0.22^b^			
4-Year College Graduate	199 (23.3%)	2.55 (0.8)	− 0.12			
Some School Beyond College	26 (3.0%)	2.53 (0.9)	− 0.21			
Graduate or Professional Degree	255 (29.8%)	2.69 (0.8)	Reference			
April Physical Health						
Excellent	103 (12.0%)	2.48 (0.9)	0.09	391 (50.4%)	2.02 (0.6)	Reference
Very Good	292 (34.1%)	2.54 (0.8)	0.00	286 (36.9%)	2.29 (0.7)	0.07
Good	298 (34.8%)	2.66 (0.8)	Reference	80 (10.3%)	2.37 (0.7)	0.01
Fair	130 (15.2%)	2.81 (0.8)	0.10	16 (2.1%)	2.79 (0.9)	0.45^b^
Poor	33 (3.9%)	3.15 (0.8)	0.24	3 (0.4%)	2.93 (0.5)	−
Prior Perceived Mental Health						
Excellent	146 (17.1%)	2.06 (0.7)	− 0.38^c^	328 (42.1%)	1.90 (0.6)	Reference
Very Good	277 (32.4%)	2.48 (0.7)	Reference	274 (35.1%)	2.21 (0.6)	0.26^c^
Good	266 (31.1%)	2.78 (0.7)	0.26^c^	130 (16.7%)	2.51 (0.6)	0.54^c^
Fair	128 (15.0%)	3.13 (0.7)	0.61^c^	40 (5.1%)	2.79 (0.9)	0.74^c^
Poor	39 (4.6%)	3.49 (0.8)	0.94^c^	8 (1.0%)	2.86 (0.6)	−
May Lose Job						
No	132 (15.4%)	2.64 (0.8)	− 0.06			
Yes	44 (5.1%)	3.07 (0.8)	0.24^a^			
Not Applicable	682 (79.5%)	2.61 (0.8)	Reference			
May Employment Status						
Working	438 (51.6%)	2.61 (0.8)	Reference			
Unemployed	129 (15.2%)	2.78 (0.7)	0.03			
Student	100 (11.8%)	2.94 (0.8)	0.07			
Retired, Disabled or Homemaker	182 (21.4%)	2.44 (0.8)	− 0.11			
April School Closure						
School Did Not Close				29 (3.7%)	2.17 (0.7)	0.05
School Did Close				710 (91.3%)	2.17 (0.7)	Reference
Not Applicable				39 (5.0%)	2.12 (0.7)	− 0.04
Essential Worker in Family						
No	581 (68.4%)	2.63 (0.8)	Reference			
Yes	268 (31.6%)	2.67 (0.8)	− 0.14			
May Health Impact						
No	675 (78.7%)	2.61 (0.8)	Reference	639 (82.0%)	2.13 (0.7)	Reference
Yes	183 (21.3%)	2.76 (0.8)	− 0.01	140 (18.0%)	2.35 (0.8)	0.04
May Financial Impact						
No	570 (66.4%)	2.56 (0.8)	Reference	570 (73.2%)	2.11 (0.6)	Reference
Yes	288 (33.6%)	2.80 (0.8)	0.11	209 (26.8%)	2.34 (0.7)	0.12^a^
May Exposure						
No	770 (89.7%)	2.62 (0.8)	Reference	735 (94.4%)	2.15 (0.7)	Reference
Yes, diagnosis positive	13 (1.5%)	3.13 (0.9)	0.12	10 (1.3%)	3.13 (1.1)	0.45^a^
Yes, symptoms only	48 (5.6%)	2.89 (0.9)	0.13	28 (3.6%)	2.21 (0.6)	− 0.04
Yes, tested positive	27 (3.1%)	2.58 (0.9)	− 0.12	6 (0.8%)	2.67 (0.7)	−
May Infected						
Yes, has positive test	1 (0.1%)	1.50 (NA)	−	0 (0.0%)	NA	−
Yes, medical diagnosis, no test	7 (0.8%)	3.13 (0.8)	−	2 (0.3%)	3.15 (0.4)	−
Yes, symptoms, no diagnosis	96 (11.2%)	2.92 (0.8)	0.16^a^	39 (5.0%)	2.22 (0.7)	− 0.20
No symptoms or signs	753 (87.9%)	2.60 (0.8)	Reference	737 (94.7%)	2.16 (0.7)	Reference
Family Member Dx						
No	798 (93.1%)	2.63 (0.8)	Reference	728 (93.7%)	2.15 (0.7)	Reference
Yes	59 (7.1%)	2.81 (0.7)	0.15	49 (6.3%)	2.48 (0.7)	0.22*
2-Week Symptom Count						
None	502 (58.5%)	2.52 (0.8)	Reference	573 (73.6%)	2.13 (0.7)	Reference
One	194 (22.6%)	2.71 (0.8)	0.11	156 (20.0%)	2.24 (0.7)	0.07
Two	81 (9.4%)	2.93 (0.8)	0.85	28 (3.6%)	2.41 (0.9)	0.24
Three or More	81 (9.4%)	2.97 (0.7)	0.04	22 (2.8%)	2.34 (0.7)	0.11

Note: N = 858 adults and 780 children assessed via parent report. Current Mood factor scores range from 1 to 5. Adjusted associations were estimated via multiple linear regression. Beta values were not estimated for cells with less than ten participants.

a = p < .05.

b = p < .01.

c = p < .001.

**Table 2:** Mood, COVID Worries, demographic characteristics, and COVID impact indicators across May life change stress subtypes.

		Adult (Self Report)				Child (Parent Report)			
	
		Life Change Stress Subtype				Life Change Stress Subtype			
			
		Lower Social/Lower Economic (1) *n* = 457	Higher Social/Higher Economic (2) *n* = 598	Lower Social/Higher Economic (3) *n* = 325		Lower Social/Lower Economic (1) *n* = 420	Higher Social/Higher Economic (2) *n* = 557	Intermediate Social/Lower Economic (3) *n* = 887	
							
		*M (SD)* or %	*M (SD)* or %	*M (SD)* or %	Post-hoc	*M (SD)* or %	*M (SD)* or %	*M (SD)* or %	Post-hoc

Factor Scores	COVID Worries (May)	2.7 (0.6)	* * * 3.2 (0.7)	2.8 (0.7)	2 > 3>1	2.0 (0.6)	* * * 2.5 (0.7)	2.2 (0.6)	2 > 3>1
	Prior Mood (April)	2.1 (0.6)	* * * 2.4 (0.8)	2.2 (0.7)	2 > 3>1	1.8 (0.6)	* * * 2.0 (0.7)	1.9 (0.6)	2 > 1 = 3
	Current Mood (May)	2.3 (0.6)	* * * 3.1 (0.7)	2.5 (0.7)	2 > 3>1	1.9 (0.5)	* * * 2.7 (0.7)	2.2 (0.5)	2 > 3>1
	Current Mood (November)	2.3 (0.7)	* * * 3.0 (0.8)	2.5 (0.7)	2 > 3>1	1.9 (0.6)	* * * 2.4 (0.7)	2.1 (0.6)	2 > 3>1
Background	Gender		–				–		
	Male	40.5%	42.6%	44.6%		53.3%	52.1%	56.6%	
	Female	58.2%	56.0%	54.8%		46.0%	47.8%	43.4%	
	Other	1.3%	1.3%	0.6%		0.7%	0.2%	0.0%	
	Age		* * *						
	Under 30	23.0%	40.0%	38.2%	2 = 3 > 1				
	30–49	41.4%	41.0%	35.7%	NS				
	Over 50	35.7%	19.1%	26.2%	1 > 3>2				
	Child Age						* *		
	5 and Under					15.7%	20.3%	20.4%	NS
	6–13					60.0%	64.8%	60.7%	NS
	14–17					21.9%	13.3%	18.4%	1 = 3 > 2
	18 and Older					2.4%	1.6%	0.5%	NS
	Race/ethnicity		* * *				–		
	Asian	6.6%	11.2%	15.7%	3 > 2>1	5.0%	2.9%	3.6%	
	Black	5.0%	5.7%	8.0%	NS	6.7%	4.1%	4.1%	
	Hispanic	7.9%	8.2%	11.1%	NS	5.0%	7.7%	8.2%	
	Other	4.2%	5.4%	4.6%	NS	3.1%	3.8%	3.1%	
	White	76.4%	69.6%	60.6%	1 > 2>3	80.2%	81.5%	81.1%	
COVID Impact	School Closed School Did Close	8.8%	* * * 20.3%	19.8%	2 = 3 > 1	87.3%	– 88.6%	89.8%	
	School Did Not Close	1.3%	1.8%	2.5%	NS	5.7%	6.1%	6.1%	
	Not Applicable	89.9%	77.9%	77.8%	1 > 2 = 3	6.9%	5.2%	4.1%	
	Job Loss		* * *						
	Job Prior, Still Working	54.6%	45.5%	34.9%	1 > 2>3				
	Job Prior, Not Still Working	13.6%	25.7%	28.4%	2 = 3 > 1				
	Did Not Have Job Prior	31.8%	28.9%	36.7%	3 > 1 = 2				

*Note*. *N* = 1380 adults and *N* = 1173 children assessed via parent report. Chi-square tests and one-way analysis of variance were conducted to test group differences. Variables for prior mood and race were assessed at the April timepoint and current mood was assessed at both the May and November timepoints. All other variables were assessed at the May timepoint.
